# Application and discoveries of metabolomics and proteomics in the study of female infertility

**DOI:** 10.3389/fendo.2023.1315099

**Published:** 2024-01-10

**Authors:** Junhua Shi, Xingjie Wu, Haiou Qi, Xin Xu, Shihao Hong

**Affiliations:** ^1^ Nursing Department, Sir Run Run Shaw Hospital, School of Medicine, Zhejiang University, Hangzhou, China; ^2^ Department of Obstetrics, Hangzhou Medical College Affiliated Lin’an People’s Hospital, The First People’s Hospital of Hangzhou Lin’an District, Hangzhou, Zhejiang, China; ^3^ Department of Obstetrics and Gynecology, Sir Run Run Shaw Hospital, School of Medicine, Zhejiang University; Key Laboratory of Reproductive Dysfunction Management of Zhejiang Province, Hangzhou, China

**Keywords:** female infertility, Mendelian randomization (MR), metabolomics, proteomics, bioinformatics

## Abstract

**Introduction:**

Female infertility is defined as the absence of clinical pregnancy after 12 months of regular unprotected sexual intercourse.

**Methods:**

This study employed metabolomics and proteomics approaches to investigate the relationship between metabolites and proteins and female infertility. The study used metabolomics and proteomics data from the UK Biobank to identify metabolites and proteins linked to infertility.

**Results:**

The results showed that GRAM domain-containing protein 1C and metabolites fibrinogen cleavage peptides ADpSGEGDFXAEGGGVR and 3-Hydroxybutyrate had a positive correlation with infertility, whereas proteins such as Interleukin-3 receptor subunit alpha, Thrombospondin type-1 domain-containing protein 1, Intestinal-type alkaline phosphatase, and platelet and endothelial cell adhesion molecule 1 exhibited a negative correlation. These findings provide new clues and targets for infertility diagnosis and treatment. However, further research is required to validate these results and gain a deeper understanding of the specific roles of these metabolites and proteins in infertility pathogenesis.

**Discussion:**

In conclusion, metabolomics and proteomics techniques have significant application value in the study of infertility, allowing for a better understanding of the biological mechanisms underlying infertility and providing new insights and strategies for its diagnosis and treatment. These research findings provide a crucial biological mechanistic basis for early infertility screening, prevention, and treatment.

## 1 Introduction

Female infertility, also known as female sterility, is when clinical pregnancy cannot be established after 12 months of regular unprotected intercourse (1). Infertility affects approximately 8%–12% of couples of reproductive age worldwide (2). While male factors are the sole cause of infertility in 20%–30% of cases, they contribute to 50% of overall infertility cases. The most prevalent type of female infertility worldwide is secondary infertility caused by reproductive tract infections. Many women around the world are infertile, which is a worldwide problem (3).Three main factors that influence the natural conception rate include the period of intention to conceive, the age of the female partner, and infertility related to medical conditions (4). Factors affecting male and female fertility include gonadal dysfunction, hyperprolactinemia, ciliary dysfunction, cystic fibrosis, infections, systemic disorders, and lifestyle/disease-related factors. Meanwhile, premature ovarian insufficiency, polycystic ovary syndrome, endometriosis, uterine fibroids, and endometrial polyps can all lead to female infertility (5). In addition, endocrine-disrupting chemicals may also be linked to infertility by interfering with metabolism (6).

Metabolomics and proteomics are multidisciplinary sciences investigating the metabolites and proteins within organisms (7). Metabolomics is the study of metabolites within an organism to reveal the activity of metabolic pathways, changes in metabolite concentrations, and interactions between metabolites, ultimately revealing the organism’s metabolic state and physiological functions (8, 9). Proteomics, on the other hand, studies the composition, structure, and function of proteins within organisms to elucidate their roles and regulatory mechanisms in cells and organisms (10). Since metabolites are typically the end products of protein metabolism, and proteins are involved in regulating metabolic pathways and the generation of metabolites, these two techniques complement one other, providing a more comprehensive understanding of an organism’s metabolic state and functionality (11, 12).

Metabolomics technology can analyze the metabolic profiles of infertility patients and normal populations to identify specific metabolites associated with infertility and reveal potential infertility mechanisms. By identifying these metabolites, an in-depth understanding of metabolic abnormalities in infertility is gained, laying the groundwork for diagnosis and treatment (13). Furthermore, metabolomics can examine alterations in metabolic pathways, revealing the mechanisms of metabolic dysfunction in infertility and potentially identifying new treatment targets (14). Any possible identified biomarkers of infertility can be used for diagnosis and prediction. Additionally, metabolomics can assist in evaluating the efficacy of infertility treatment drugs by analyzing their effects on the metabolic spectrum and predicting treatment outcomes (15). Overall, metabolomics has significant value in screening, identifying, diagnosing, and evaluating infertility treatment (13). Proteomics is also significant in infertility studies. By identifying proteins linked to infertility, a better understanding of these diseases can be gained, providing better guidelines for early diagnosis and treatment. Future research should identify specific proteins related to these diseases and embryonic development outcomes and establish their reproducibility and reliability (16). It is intended that by identifying common proteins among different races, ages, and regions, advances to women’s overall health worldwide may be achieved. Therefore, metabolomics and proteomics play crucial roles in early screening, prevention, and understanding of the potential biological mechanisms of infertility treatment (17).

Mendelian randomization (MR) evaluates causality by utilizing natural genetic variation. This technique stimulates the effect of a randomized controlled trial by using human genetic variation as a natural random tool to examine whether a factor has a causal effect on a disease or other biological characteristics (18). In this study, we used metabolomics and proteomics data for MR analysis, comprehensively investigating the causal effects of 975 metabolites and 4490 proteins on the onset of infertility. Our goal was to study the biological mechanisms of infertility at the genetic and protein levels, focusing on revealing the etiology of metabolic-related diseases and deepening understanding of their biological processes (19).

## 2 Materials and methods

### 2.1 Metabolomics and proteomics GWAS data

Metabolite GWAS data used in this study were obtained from the open GWAS website (https://gwas.mrcieu.ac.uk/). The 975 metabolites retrieved contained 452 human blood metabolites, 150 metabolites of human immune system symptoms, 123 circulating metabolites, and 249 metabolic traits, which were identified in 115,078 participants with nearly 12 million SNPs (Single Nucleotide Polymorphism) from the UK Biobank using the Nightingale Health assay. Four continuous sources of overlapping data were eliminated, and 975 metabolites were retained (20–22). The proteomics data in this study contained a total of 4490 distinct data from various proteins from three separate sets of studies (23–25).

### 2.2 Outcome GWAS data

The Infertility GWAS data in this study came from the Finngen population database, a large-scale human genomics database incorporating genetic, health, and clinical data. This database serves as a valuable data resource and analytical tool for genetic research and precision medicine, which helps elucidate the genetic basis of diseases and develop individualized medicine(https://risteys.finregistry.fi/endpoints/N14_FEMALEINFERT). In this data representation, female infertility, defined as the inability of female to get pregnant after a specified period of unprotected intercourse, ICD10-N97.

### 2.3 Selection of instrumental variables

To meet the experimental hypothesis of Mendelian randomization [MR], the instrumental variable selection should have strong relevance in the following aspects: 1) Instrumental variables should have a strong correlation with the outcome variable [dependent variable], implying that instrumental variables should have a large explanatory power for the variation of the outcome variable. As a result, the SNPs effectively represent the variation in the outcome variable; 2) Unrelatedness: instrumental variables should be unrelated to other factors that influence variation in the outcome variable. This was done to prevent instrumental variables associated with other potential confounding variables that could generate endogeneity problems; 3) Exclusivity: instrumental variables should only affect the outcome variable through their influence on the dependent variable rather than via additional pathways. This ensures that the instrumental variables only impact the outcome variable via causal pathways without interference from other factors. Therefore, we defined strict standards for the inclusion of instrumental variables, with a whole-genome significant level [P-value < 5× 10^−8^] and no linkage disequilibrium [LD] with other SNPs [r2< 0.01] as the instrumental variables for these metabolites and proteins.

### 2.4 Statistical analysis

This study used inverse variance-weighted [IVW] meta-analysis to investigate and assess the causal relationships between exposure, intermediate, and outcome (26). The IVW method is the primary MR analysis method used for combining the Wald ratios of individual SNPs (27). The estimates may be biased since the methodology assumes that all variants are effective instruments and that the instrumental variables may exhibit directional pleiotropy (28). Cochran’s Q value was used to determine whether the analysis was heterogeneous. Moreover, we employed forest plots to demonstrate the correlations between SNP exposure association and outcome association, limit the effect of errors, increase experimental reliability and accuracy, and measure the contribution of each instrumental variable to the overall causal estimate.

## 3 Results

### 3.1 Inclusion of study variables

**Figure 1:**
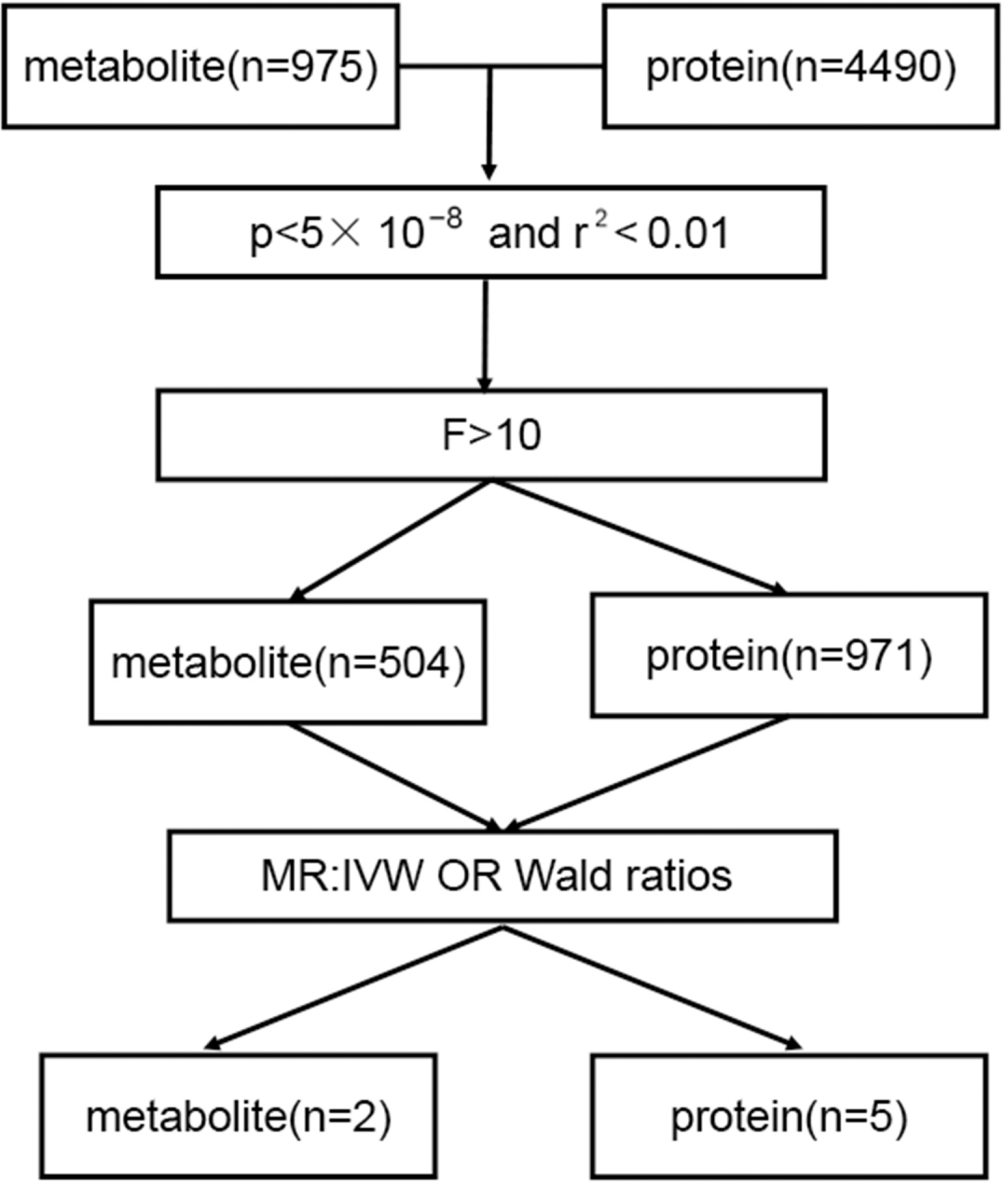
Flow chart of this study.

After a rigorous screening of instrumental variables, 504 metabolites and 971 proteins were included in the study. In the MR study, 5 proteins and 2 metabolites showed potential causal relationships. fibrinogen cleavage peptides ADpSGEGDFXAEGGGVR and 3-Hydroxybutyrate were among the metabolites included, while the proteins were GRAM domain-containing protein 1C, Interleukin-3 receptor subunit alpha, Thrombospondin type-1 domain-containing protein 1, Intestinal-type alkaline phosphatase, and platelet and endothelial cell adhesion molecule 1. **Figure 1** depicts the flowchart.

### 3.2 Protein-mediated MR results for infertility

**Figure 2:**
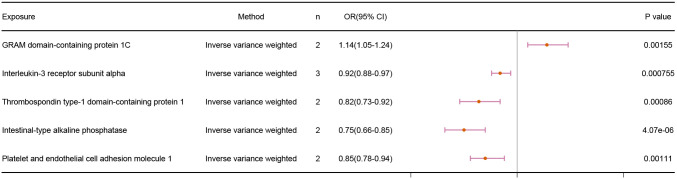
Association results of MR analyses with 5 Protein and Infertility.

Our findings revealed a positive correlation between GRAM domain-containing protein 1C and infertility, demonstrating that GRAM domain-containing protein 1C is a risk factor for infertility. The other 4 proteins, on the other hand, displayed a negative correlation with infertility. The IVW results were as follows: GRAM domain-containing protein 1C (p = 1.55E-03; OR 95% CI = 1.14(1.05,1.24)), Interleukin-3 receptor subunit alpha (p = 7.55E-04; OR 95% CI = 0.92(0.88,0.97)), Thrombospondin type-1 domain-containing protein 1 (p = 8.60E-04; OR 95% CI = 0.82(0.73,0.92)), Intestinal-type alkaline phosphatase (p =4.07E-06; OR 95% CI = 0.75(0.66,0.85)), and platelet and endothelial cell adhesion molecule 1 (p = 1.11E-03; OR 95% CI = 0.85(0.78,0.94)). The results are shown in **Figure 2**.

### 3.3 Metabolite-mediated MR results for infertility

**Figure 3:**
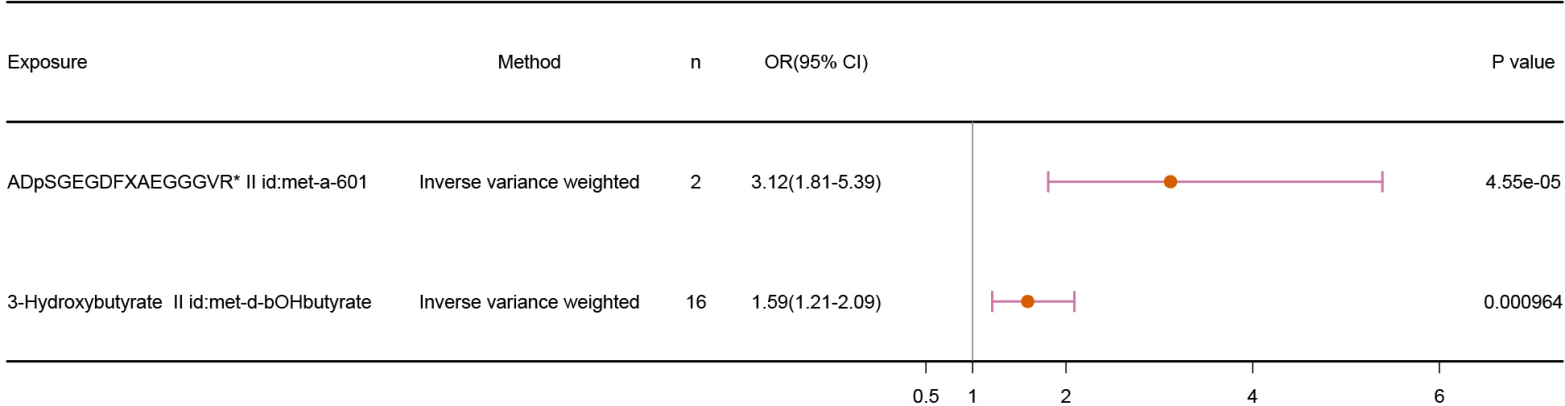
Association results of MR analyses with 2 Metabolites and Infertility.

Our results demonstrated a positive relationship between fibrinogen cleavage peptides ADpSGEGDFXAEGGGVR and 3-Hydroxybutyrate and infertility, suggesting the metabolites as risk factors for infertility. The IVW results, shown in **Figure 3**, are as follows: fibrinogen cleavage peptides ADpSGEGDFXAEGGGVR (p = 4.55E-05; OR 95% CI = 3.12(1.81,5.39)) and 3-Hydroxybutyrate (p = 9.64E-04; OR 95% CI = 1.59(1.21,2.09)).

In the subsequent sensitivity analysis, the P-values and Cochran’s Q P-values for all included metabolites and proteins were above 0.05, indicating no outliers in our data and the robustness of our results. The sensitivity analysis results can be found in the **Supplementary Materials**. In the subsequent leave-one-out analysis, our results remained stable even with gradual removal of instrumental variables. MR-Egger analysis could not be performed to test the pleiotropy of 4 proteins and 1 metabolites because the IVs were less than three SNPs. Detailed analysis information can be found in the **Supplementary Table 1** and the relevant biological functions or pathways of these targeted proteins and metabolites were list in the **Supplementary Table 2**.

## 4 Discussion

Our study used large-scale metabolomics and proteomics data for MR analysis to discover potential relationships between metabolites, proteins, and female infertility. We identified several metabolites and proteins that may be linked to female infertility, providing insight into its biological mechanisms. Female infertility refers to a woman’s inability to conceive under normal sexual conditions and within one year of attempting to do so (29). Ovulation disorders, uterine abnormalities, tubal obstruction, and ovarian dysfunction are the most common causes (30, 31). The most prevalent cause is ovulation abnormalities, which can caused by polycystic ovary syndrome, ovarian dysfunction, thyroid dysfunction, and other factors (32). Infection, surgery, or congenital abnormalities can induce tubal obstruction. Ovarian dysfunction includes premature ovarian failure and ovarian cysts. Female infertility symptoms may include irregular, abnormally lengthy, or short menstrual cycles, and difficulty ovulating (33). Proteomics and metabolomics are two significant omics technologies for studying and diagnosing female infertility. Proteomics compares protein spectra between infertility patients and normal populations, detects protein expression differences associated with infertility, and identifies protein biomarkers for infertility diagnosis and treatment. Metabolomics examines the metabolite spectra of infertility patients and normal populations, identifies metabolites related to infertility, and employs them as biomarkers for infertility assessment and treatment. These two technologies can offer vital information on the mechanisms and individual characteristics of infertility, laying the groundwork for infertility research and treatment (34, 35). By applying these omics technologies, we can gain a deeper understanding of the pathogenesis of infertility, discover new biomarkers, and provide new insights and strategies for infertility diagnosis and treatment. Furthermore, these omics technologies can also help evaluate the effectiveness of infertility treatments and offer a foundation for more personalized treatments.

3-hydroxybutyric acid (3-HB) is a metabolite commonly associated with fatty acid metabolism and the synthesis of ketone bodies (36). Although no conclusive data links 3-hydroxybutyric acid (3-HB) directly to infertility, it may be indirectly associated with certain aspects of infertility. As a metabolite, 3-HB may be indirectly linked to certain aspects of infertility. 3-HB is a ketone body primarily synthesized by the liver, and its levels can rise in specific conditions such as fasting, prolonged exercise, and diabetes (37, 38). Infertility can be linked to metabolic disorders and hormonal imbalances; variations in 3-HB levels can reflect these changes. For example, 3-HB levels are often elevated in diabetic patients, which is a factor contributing to infertility. Additionally, 3-HB may be related to ovarian function (39, 40). The ovaries are essential organs in the female reproductive system responsible for ovulation and hormone secretion. Some studies have suggested a possible link between 3-HB and ovarian function (41). For instance, one study found that 3-HB levels were associated with ovarian reserve, with higher levels indicating better ovarian reserve. Ovarian reserve is a significant predictor of female fertility (42). Therefore, 3-HB may be related to ovarian dysfunction in female infertility. PECAM-1 (Platelet Endothelial Cell Adhesion Molecule-1) is a protein implicated in infertility. PECAM-1 is widely present in the reproductive system, including the ovaries, endometrium, and fallopian tubes. Some studies have found associations between PECAM-1 and infertility factors such as ovulation disorders, uterine abnormalities, and tubal obstruction (43). For example, PECAM-1 deficiency may lead to follicle development and ovulation disorders. Patients with endometriosis and endometrial cancer generally have an increased expression of PECAM-1. Additionally, elevated PECAM-1 expression has been identified in individuals with fallopian tube cancer and tubal tuberculosis (44). These findings suggest that PECAM-1 may play a role in the onset and progression of infertility. Further research on the relationship between PECAM-1 and infertility should aid in understanding the etiology and treatment of infertility. Unfortunately, research into the interaction between other metabolites and proteins and infertility is limited, and no clear link with infertility has been identified. Further investigation into the role of these substances in infertility is required.

This article has several strengths:

1. The article provides detailed information about the definition of female infertility, its global prevalence, and potential causes and influencing variables, offering readers a thorough grasp of the condition.

2. The article utilized Mendelian randomization (MR) analysis to investigate potential causal relationships between metabolites, proteins, and infertility using large-scale metabolomics and proteomics data.

3. The article provides detailed statistical analysis methods and results, including instrument variable selection, statistical analysis methods, and sensitivity analysis, enhancing the reliability and feasibility of the study.

However, there are some limitations to this article:

1. The article does not provide detailed information about other potential causes and influencing factors of infertility, such as genetic and environmental factors, which may limit a comprehensive understanding of the etiology of infertility.

2. The study results are based only on existing metabolomics and proteomics data, which may be limited by sample size and data quality, requiring further research to validate and replicate these findings.

## 5 Conclusion

Based on the integrated findings from metabolomics and proteomics research, several potential relationships have been identified between metabolites, proteins, and female infertility. These findings provide new clues and targets for infertility diagnosis and treatment.

## Data availability statement

The original contributions presented in the study are included in the article/**Supplementary Material**. Further inquiries can be directed to the corresponding author.

## Author contributions

JS: Data curation, Writing – original draft, Writing – review & editing. XW: Writing – review & editing. HQ: Data curation, Writing – original draft. XX: Writing – original draft. SH: Supervision, Validation, Writing – review & editing.

## References

[1] Vander Borght M Wyns C. Fertility and infertility: Definition and epidemiology. Clin Biochem (2018) 62:2–10. doi: 10.1016/j.clinbiochem.2018.03.012 29555319

[2] Bala R Singh V Rajender S Singh K. Environment, lifestyle, and female infertility. Reprod Sci (2021) 28(3):617–38. doi: 10.1007/s43032-020-00279-3 32748224

[3] Dompe C Kulus M Stefanska K Kranc W Chermula B Bryl R. Human granulosa cells-stemness properties, molecular cross-talk and follicular angiogenesis. Cells (2021) 10(6). doi: 10.3390/cells10061396 PMC8229878 34198768

[4] Haller-Kikkatalo K Salumets A Uibo R. Review on autoimmune reactions in female infertility: antibodies to follicle stimulating hormone. Clin Dev Immunol (2012) 2012:762541. doi: 10.1155/2012/762541 22007255 PMC3189473

[5] Wojsiat J Korczynski J Borowiecka M Zbikowska HM. The role of oxidative stress in female infertility and in *vitro* fertilization. Postepy Hig Med Dosw (Online). (2017) 71(0):359–66. doi: 10.5604/01.3001.0010.3820 28513460

[6] Guo W Yue J Zhao Q Zhang L Lu S. Comparison of 17beta-estradiol adsorption on corn straw- and dewatered sludge-biochar in aqueous solutions. Molecules. (2022) 27(8). doi: 10.3390/molecules27082567 PMC9030855 35458764

[7] Rozanova S Barkovits K Nikolov M Schmidt C Urlaub H Marcus K. Quantitative mass spectrometry-based proteomics: an overview. Methods Mol Biol (2021) 2228:85–116. doi: 10.1007/978-1-0716-1024-4_8 33950486

[8] Schrimpe-Rutledge AC Codreanu SG Sherrod SD McLean JA. Untargeted metabolomics strategies-challenges and emerging directions. J Am Soc Mass Spectrom (2016) 27(12):1897–905. doi: 10.1007/s13361-016-1469-y PMC5110944 27624161

[9] Wang R Li B Lam SM Shui G. Integration of lipidomics and metabolomics for in-depth understanding of cellular mechanism and disease progression. J Genet Genomics (2020) 47(2):69–83. doi: 10.1016/j.jgg.2019.11.009 32178981

[10] Zhang Z Wu S Stenoien DL Pasa-Tolic L. High-throughput proteomics. Annu Rev Anal Chem (Palo Alto Calif). (2014) 7:427–54. doi: 10.1146/annurev-anchem-071213-020216 25014346

[11] Rotello RJ Veenstra TD. Mass spectrometry techniques: principles and practices for quantitative proteomics. Curr Protein Pept Sci (2021) 22(2):121–33. doi: 10.2174/1389203721666200921153513 32957902

[12] Guijas C Montenegro-Burke JR Warth B Spilker ME Siuzdak G. Metabolomics activity screening for identifying metabolites that modulate phenotype. Nat Biotechnol (2018) 36(4):316–20. doi: 10.1038/nbt.4101 PMC5937131 29621222

[13] Mehrparavar B Minai-Tehrani A Arjmand B Gilany K. Metabolomics of male infertility: A new tool for diagnostic tests. J Reprod Infertil. (2019) 20(2):64–9. PMC6486563 31058049

[14] Minai-Tehrani A Jafarzadeh N Gilany K. Metabolomics: a state-of-the-art technology for better understanding of male infertility. Andrologia. (2016) 48(6):609–16. doi: 10.1111/and.12496 26608970

[15] Wagner AO Turk A Kunej T. Towards a multi-omics of male infertility. World J Mens Health (2023) 41(2):272–88. doi: 10.5534/wjmh.220186 PMC10042660 36649926

[16] Agarwal A Durairajanayagam D Halabi J Peng J Vazquez-Levin M. Proteomics, oxidative stress and male infertility. Reprod BioMed Online (2014) 29(1):32–58. doi: 10.1016/j.rbmo.2014.02.013 24813754

[17] Fabozzi G Verdone G Allori M Cimadomo D Tatone C Stuppia L. Personalized nutrition in the management of female infertility: new insights on chronic low-grade inflammation. Nutrients (2022) 14(9). doi: 10.3390/nu14091918 PMC9105997 35565885

[18] Bowden J Holmes MV. Meta-analysis and Mendelian randomization: A review. Res Synth Methods (2019) 10(4):486–96. doi: 10.1002/jrsm.1346 PMC6973275 30861319

[19] Birney E. Mendelian randomization. Cold Spring Harb Perspect Med (2022) 12(4). doi: 10.1101/cshperspect.a041302 PMC9121891 34872952

[20] Shin SY Fauman EB Petersen AK Krumsiek J Santos R Huang J. An atlas of genetic influences on human blood metabolites. Nat Genet (2014) 46(6):543–50. doi: 10.1038/ng.2982 PMC4064254 24816252

[21] Roederer M Quaye L Mangino M Beddall MH Mahnke Y Chattopadhyay P. The genetic architecture of the human immune system: a bioresource for autoimmunity and disease pathogenesis. Cell. (2015) 161(2):387–403. doi: 10.1016/j.cell.2015.02.046 25772697 PMC4393780

[22] Kettunen J Demirkan A Wurtz P Draisma HH Haller T Rawal R. Genome-wide study for circulating metabolites identifies 62 loci and reveals novel systemic effects of LPA. Nat Commun (2016) 7:11122. doi: 10.1038/ncomms11122 27005778 PMC4814583

[23] Sun BB Maranville JC Peters JE Stacey D Staley JR Blackshaw J. Genomic atlas of the human plasma proteome. Nature (2018) 558(7708):73–9. doi: 10.1038/s41586-018-0175-2 PMC6697541 29875488

[24] Folkersen L Fauman E Sabater-Lleal M Strawbridge RJ Franberg M Sennblad B. Mapping of 79 loci for 83 plasma protein biomarkers in cardiovascular disease. PloS Genet (2017) 13(4):e1006706. doi: 10.1371/journal.pgen.1006706 28369058 PMC5393901

[25] Suhre K Arnold M Bhagwat AM Cotton RJ Engelke R Raffler J. Connecting genetic risk to disease end points through the human blood plasma proteome. Nat Commun (2017) 8:14357. doi: 10.1038/ncomms14357 28240269 PMC5333359

[26] Zoccali C. The challenge of Mendelian randomization approach. Curr Med Res Opin (2017) 33(sup3):5–8. doi: 10.1080/03007995.2017.1378514 28952387

[27] Burgess S Davey Smith G Davies NM Dudbridge F Gill D Glymour MM. Guidelines for performing Mendelian randomization investigations: update for summer 2023. Wellcome Open Res (2019) 4:186. doi: 10.12688/wellcomeopenres.15555.3 32760811 PMC7384151

[28] Burgess S Thompson SG. Erratum to: Interpreting findings from Mendelian randomization using the MR-Egger method. Eur J Epidemiol. (2017) 32(5):391–2. doi: 10.1007/s10654-017-0276-5 PMC6828068 28664250

[29] Li HQ Pan XL Su NJ Lu XP Chen JQ Chen XW. Retrospective analysis: The application of human menopausal gonadotropin combined with letrozole for IUI in patients undergoing artificial insemination by husband due to unexplained or mild male factors. Front Endocrinol (Lausanne). (2022) 13:1038433. doi: 10.3389/fendo.2022.1038433 36605946 PMC9810010

[30] Practice Committee of the American Society for Reproductive Medicine. Electronic address aao, Practice Committee of the American Society for Reproductive M. Fertility evaluation of infertile women: a committee opinion. Fertil Steril. (2021) 116(5):1255–65. doi: 10.1016/j.fertnstert.2021.08.038 34607703

[31] Xu W You Y Yu T Li J. Insights into modifiable risk factors of infertility: A mendelian randomization study. Nutrients. (2022) 14(19). doi: 10.3390/nu14194042 PMC9572512 36235694

[32] Sheikhian M Loripoor M Ghorashi Z Safdari-Dehcheshmeh F. Relation between sexual function, perceived social support, and adherence to treatment with infertility factor in women: A cross-sectional study. Int J Reprod Biomed (2023) 21(7):557–66. doi: 10.18502/ijrm.v21i7.13893 PMC10505696 37727393

[33] Wang X Zhou R Lu X Dai S Liu M Jiang C. Identification of nonfunctional PABPC1L causing oocyte maturation abnormalities and early embryonic arrest in female primary infertility. Clin Genet (2023). doi: 10.1111/cge.14425 37723834

[34] Xia Q Yu L Song J Sun Z. The role of acupuncture in women with advanced reproductive age undergoing in *vitro* fertilization-embryo transfer: A randomized controlled trial and follicular fluid metabolomics study. Med (Baltimore). (2023) 102(36):e34768. doi: 10.1097/MD.0000000000034768 PMC10489312 37682195

[35] Wei Y Zhang Z Zhang Y Li J Ruan X Wan Q. Nontargeted metabolomics analysis of follicular fluid in patients with endometriosis provides a new direction for the study of oocyte quality. MedComm (2020) (2023) 4(3):e302. doi: 10.1002/mco2.302 37265938 PMC10229744

[36] Ma L Zhang Z Li J Yang X Fei B Leung PHM. A new antimicrobial agent: poly (3-hydroxybutyric acid) oligomer. Macromol Biosci (2019) 19(5):e1800432. doi: 10.1002/mabi.201800432 30951260

[37] Hsu TT Leiske DL Rosenfeld L Sonner JM Fuller GG. 3-Hydroxybutyric acid interacts with lipid monolayers at concentrations that impair consciousness. Langmuir. (2013) 29(6):1948–55. doi: 10.1021/la304712f 23339286

[38] Deemer SE Plaisance EP Martins C. Impact of ketosis on appetite regulation-a review. Nutr Res (2020) 77:1–11. doi: 10.1016/j.nutres.2020.02.010 32193016

[39] Soto-Mota A Norwitz NG Clarke K. Why a d-beta-hydroxybutyrate monoester? Biochem Soc Trans (2020) 48(1):51–9. doi: 10.1042/BST20190240 PMC7065286 32096539

[40] Moller N. Ketone body, 3-hydroxybutyrate: minor metabolite - major medical manifestations. J Clin Endocrinol Metab (2020) 105(9). doi: 10.1210/clinem/dgaa370 32525972

[41] Huang D Hu H Chang L Liu S Liang J Song Y. Chinese medicine Bazi Bushen capsule improves lipid metabolism in ovariectomized female ApoE-/- mice. Ann Palliat Med (2020) 9(3):1073–83. doi: 10.21037/apm-20-906 32434357

[42] RoyChoudhury S Mishra BP Khan T Chattopadhayay R Lodh I Datta Ray C. Serum metabolomics of Indian women with polycystic ovary syndrome using (1)H NMR coupled with a pattern recognition approach. Mol Biosyst (2016) 12(11):3407–16. doi: 10.1039/C6MB00420B 27714060

[43] Saragih H Zilian E Jaimes Y Paine A Figueiredo C Eiz-Vesper B. PECAM-1-dependent heme oxygenase-1 regulation via an Nrf2-mediated pathway in endothelial cells. Thromb Haemost. (2014) 111(6):1077–88. doi: 10.1160/TH13-11-0923 24500083

[44] Kuwabara Y Katayama A Igarashi T Tomiyama R Piao H Kaneko R. Rapid and transient upregulation of CCL11 (eotaxin-1) in mouse ovary during terminal stages of follicular development. Am J Reprod Immunol (2012) 67(5):358–68. doi: 10.1111/j.1600-0897.2011.01100.x 22221885

